# A Service Evaluation Exploring Referrals Made From Primary Care to CAMHS in Children With a Potential Diagnosis of ADHD, Autism and Other Mental Health Conditions in a South London Based GP Practice

**DOI:** 10.1192/bjo.2025.10514

**Published:** 2025-06-20

**Authors:** Thusani Nanthakumaran

**Affiliations:** St George’s University of London, London, United Kingdom

## Abstract

**Aims:** This service evaluation aims to identify and assess referrals made to CAMHS at a south London based GP practice. The focus of this evaluation will be on referrals for Attention Deficit hyperactivity Disorder (ADHD), autism, anxiety and other mental health conditions such as depression and suicidal ideation. It will also aim to assess if the support available to parents and children is sufficient and if the long waiting times creates pressure on the practice.

**Methods:** This service evaluation has a cohort of 50 patients who were randomly selected through the EMIS database and had referrals to CAMHS from the practice for autism, ADHD, anxiety and other mental health conditions. The eight parameters that are being measured in this study are:

Age.

The type of mental health support that is offered in the community for the child, e.g. counselling.

The date of first referral from the GP practice.

The date whereby the referral was accepted or rejected.

If the referral was rejected, were there any more referrals?

If the referral was accepted, the number of appointments between referral and diagnosis at A&E.

If the referral was accepted, the number of appointments between referral and diagnosis at the GP.

The type of information and support given to the parents.

**Results:** In this study a total of 88 extra consultations were made at the practice or A&E with 84% of these consultations made at the GP. Many extra consultations were made at the general practice due to the long waiting times and worsening mental health whilst waiting for CAMHS input. 59% of referrals were rejected or put on a waiting list after the first referral was made. After the initial referral, 32 % of patients made extra referrals, the majority being for ADHD and autism. Rejected referrals for ADHD were the highest at 14% of the total cohort and rejections for other mental health conditions were the lowest at 4%. Some patients received support in the community before or whilst waiting for a referral such as occupational therapy, counselling and school support.

**Conclusion:** This retrospective study highlights the need for more clarity in referral criteria for GPs and in signposting support services during initial referral and diagnosis to prevent the condition of the patient getting worse.

